# A qualitative exploration to understand barriers and facilitators to daily oral PrEP uptake and sustained adherence among HIV‐negative women planning for or with pregnancy in rural Southwestern Uganda

**DOI:** 10.1002/jia2.25894

**Published:** 2022-03-24

**Authors:** Esther Cathyln Atukunda, Moran Owembabazi, Madeline Claire Pratt, Christina Psaros, Winnie Muyindike, Pooja Chitneni, Mwebesa Bosco Bwana, David Bangsberg, Jessica Elizabeth Haberer, Jeanne Marrazzo, Lynn Turner Matthews

**Affiliations:** ^1^ Mbarara University of Science and Technology Mbarara Uganda; ^2^ Division of Infectious Diseases University of Alabama at Birmingham Birmingham Alabama USA; ^3^ Behavioral Medicine Program Department of Psychiatry Massachusetts General Hospital Boston Massachusetts USA; ^4^ Harvard Medical School Boston Massachusetts USA; ^5^ Division of Infectious Diseases and General Internal Medicine Brigham and Women's Hospital Boston Massachusetts USA; ^6^ School of Public Health Oregon Health Sciences University – Portland State University Portland Oregon USA; ^7^ Department of Medicine Massachusetts General Hospital Boston Massachusetts USA

**Keywords:** adherence, HIV prevention trials, HIV prevention, LMIC, PrEP, retention

## Abstract

**Introduction:**

Antiretroviral pre‐exposure prophylaxis (PrEP) may reduce periconception and pregnancy HIV incidence among women in settings, where gender power imbalances limit HIV testing, engagement in care and HIV viral suppression. We conducted qualitative interviews to understand factors influencing periconception and pregnancy PrEP uptake and use in a cohort of women (Trial registration: NCT03832530) offered safer conception counselling in rural Southwestern Uganda, where PrEP uptake was high.

**Methods:**

Between March 2018 and January 2019, in‐depth interviews informed by conceptual frameworks for periconception risk reduction and PrEP adherence were conducted with 37 women including those with ≥80% and <80% adherence to PrEP doses measured by electronic pill cap, those who never initiated PrEP, and seven of their male partners. Content and dyadic analyses were conducted to identify emergent challenges and facilitators of PrEP use within individual and couple narratives.

**Results:**

The median age for women was 33 years (IQR 28, 35), 97% felt likely to acquire HIV and 89% initiated PrEP. Individual‐level barriers included unwillingness to take daily pills while healthy, side effects and alcohol use. Women overcame these barriers through personal desires to have control over their HIV serostatus, produce HIV‐negative children and prevent HIV transmission within partnerships. Couple‐level barriers included nondisclosure, mistrust and gender‐based violence; facilitators included shared goals and perceived HIV protection, which improved communication, sexual intimacy and emotional support within partnerships through a self‐controlled method. Community‐level barriers included multi‐level stigma related to HIV, ARVs/PrEP and serodifference; facilitators included active peer, family or healthcare provider support as women aspired to safely meet socio‐cultural expectations to conceive and preserve serodifferent relationships. Confidence in PrEP effectiveness was promoted by positive peer experiences with PrEP and ongoing HIV testing.

**Conclusions:**

Multi‐level forms of HIV‐, serodifference‐ and disclosure‐related stigma, side effects, pill burden, alcohol use, relationship dynamics, social, professional and partnership support towards adaptation and HIV risk reduction influence PrEP uptake and adherence among HIV‐negative women with plans for pregnancy in rural Southwestern Uganda. Confidence in PrEP, individually controlled HIV prevention and improved partnership communication and intimacy promoted PrEP adherence. Supporting individuals to overcome context‐specific barriers to PrEP use may be an important approach to improving uptake and prolonged use.

## 1 INTRODUCTION

The prevalence of HIV in Uganda is among the highest in the world, and the country has a total fertility rate of 6.2 children per woman [1]. At least 30–50% of men with HIV in Uganda desire children [2, 3, 4, 5, 6], and nearly half have a stable, uninfected partner, also called a serodifferent partnership [7]. Given that an estimated 33% of men in Uganda do not know their status and only 47% of those enrolled in care achieve HIV‐RNA suppression, many women face increased risks of HIV seroconversion when pursuing reproductive goals [2, 3, 4, 5, 8, 9]. While services to prevent perinatal transmission are robust for pregnant women with HIV, HIV prevention prior to pregnancy is not integrated within public‐sector reproductive healthcare.

Pre‐exposure prophylaxis (PrEP) presents an opportunity to conceive without acquiring HIV [10, 11]. Tenofovir (TFV) disoproxil fumarate/emtricitabine (TDF/FTC) as PrEP prevents HIV, is safe during pregnancy and is recommended by the WHO, CDC and the Uganda Ministry of Health for people exposed to HIV during periconception, pregnancy and postpartum periods [12, 13, 14]. However, PrEP uptake remains low due to awareness, access, perceived effectiveness, adherence challenges and HIV‐related stigma [10, 15, 16]. Motivations for PrEP use evolve with time, experience and context [17], particularly when gender norms make it challenging for a woman to insist that her partner participates in strategies to reduce sexual transmission of HIV [11, 18, 19].

We conducted a single‐arm study to evaluate uptake and factors associated with adherence to PrEP over 9 months among HIV‐exposed women planning for pregnancy in rural Uganda. In this qualitative component, we sought to explore factors affecting periconception and pregnancy PrEP uptake and adherence among women enrolled as individuals, independent of their partners’ disclosure or willingness to participate. We hope to highlight women's experiences in initiating and adhering to PrEP in order to inform periconception and pregnancy PrEP implementation for women.

## 2 METHODS

### 2.1 Research design

This qualitative study is embedded in a mixed‐methods study in rural Uganda that offered safer conception care to 131 HIV‐negative women reporting a partner with HIV or otherwise feeling vulnerable to HIV acquisition, with plans for pregnancy [20]. The parent prevention intervention was informed by conceptual frameworks to evaluate prevalence and determinants of periconception PrEP uptake and protective levels of adherence [21, 22, 23].

Women who were at least 18 years of age, HIV negative, non‐pregnant, likely to be fertile based on sexual and reproductive health history [24, 25], and reported personal or partner desire to have a child in the next year [26], with a partner she reported as living with or likely to be living with HIV (e.g. taking medicine daily, has implied that he is “sick” but has not disclosed) [27, 28], were recruited between March 2018 and January 2019 (Trial registration: NCT03832530). Eligible women lived within 60 km of the clinic, were not planning on relocating, were fluent in English or the dominant local language (Runyankole) and able to participate in the informed consent process.

Enrolled women participated in quarterly study visits over 9 months during which HIV and pregnancy testing, questionnaires and safer conception counselling sessions were conducted. Safer conception counselling sessions supported women to encourage their partners to test and initiate ART, to delay condomless sex until partner is virally suppressed or on ART for 6 months, to time condomless sex to peak fertility and/or to consider sperm washing and adoption as alternatives. Participants were eligible to initiate PrEP at any time. Quarterly adherence counselling was based on the Lifesteps adherence intervention adapted to support PrEP use [29, 30]. Adherence was determined using Wisepill, which uses wireless technology to monitor pill cap openings and, therefore, medication use [31]. The 80% adherence threshold was chosen based on protective dosing in studies available at the time of study design [29, 32, 33]; more recent data suggest that near‐daily adherence is required for protection from vaginal exposures [34]. Adherence data were used to select diverse interview participants to inform a broad description of facilitators and barriers to PrEP use.

### 2.2 Participant recruitment and enrolment

Women were purposively sampled to participate in exit in‐depth interviews from three groups (∼15 per group): (1) those who did not initiate PrEP, (2) those who initiated PrEP and took ≥80% of doses, and (3) those who took <80% of doses. Interview participants were encouraged to invite their male partners for a separate interview to explore barriers and facilitators to PrEP use and other safer conception strategies. Partners could come alone or with the female participant; seven presenting partners were consented and participated in interviews.

### 2.3 Data collection

Interview guides were developed based on conceptual frameworks for periconception risk reduction and PrEP adherence to explore constructs expected to impact PrEP uptake and adherence [21, 22, 23]. We explored *periconception* HIV transmission risk, *risk reduction* options, PrEP uptake, experiences and perceptions of *PrEP*, adherence experiences, such as dosing behaviour, circumstances around missed doses, barriers to adherence, who or what promoted adherence, how pregnancy plans influenced adherence behaviour and reasons for changes in adherence patterns over time. Interviews were conducted until no new information was obtained on HIV, PrEP and adherence. Enrolled male partners participated in a separate in‐depth interview that explored reproductive goals, experiences with HIV, understandings and use of safer conception methods, HIV serostatus disclosure, partnership dynamics and satisfaction with the study. All interviews were conducted in Runyankole, the dominant local language, and digitally recorded.

### 2.4 Data analysis

To ensure accuracy, transcripts were translated into English by two Ugandan research assistants who are fluent in both Runyankole and English. Four team members (ECA, MO, LTM and MCP) met weekly to develop a codebook through conventional content analysis [35]. Upon finalization of the codebook, transcripts were coded by two team members (MO and MCP). Five (11%) transcripts were coded by both researchers, to calculate an intercoder reliability Kappa statistic of 0.83. Analysis was organized using NVivo 12. Dyadic analysis involved the examination of emergent themes from individual and couple narratives [36]. Demographic, reproductive health, safer conception behaviours and depression [37] data of interview participants as collected at enrolment were analysed using STATA Version 13.

## 3 RESULTS

### 3.1 Participant characteristics

Thirty‐seven study participants and seven male partners were interviewed. The median age was 33 years (IQR 28, 35) for women and 35 years (IQR 31, 38) for men. Sixty‐two percent of female participants had less than secondary education, and 68% had an income less than 300,000 UGX (equivalent to ∼83 USD) in the past 3 months. Most women (63%) reported their pregnancy partner as their spouse or legal partner. Twenty‐seven percent were unemployed, 97% felt likely to acquire HIV and 89% initiated PrEP. More than 65% of women reported at least three pregnancies in their lifetime, and 38% reported depression. Twenty percent of women reported routine condom use. Most (92%) females and all men reported desire for a (partner) pregnancy “now” or “as soon as possible.” Other demographics are represented in Table 1.

**Table 1:** Characteristics of study participants

Category	Female participants (*N* = 37) *N* (%) Median (IQR)	Male partners (*N* = 7) *N* (%) Median (IQR)
Age (median, IQR)	33 (28.1, 35.4)	35 (30.6, 37.7)
Less than primary education	13 (35.1)	2 (28.6)
Less than secondary education	23 (62.2)	3 (42.9)
Unemployed	10 (27.0)	1 (14.3)
Monthly household income <300,000 UGX, past 3 months	25 (67.6)	1 (14.3)
Feeling likely to acquire HIV	36 (97.3)	N/A
Initiated PrEP	33 (89.2)	N/A
Number of pregnancies in lifetime		
0	3 (8.1)	N/A
1	3 (8.1)	
2	7 (18.9)	
≥3	24 (64.9)	
Number of children currently alive (median, IQR)	2 (0, 4)	2 (0, 4)
Depression (> 1.75: Hopkins symptom checklist)	14 (37.8)	–
Sexual partners (last 3 months)		
0	2 (5.4)	1 (14.3)
1	33 (89.2)	5 (71.4)
≥2	2 (5.4)	1 (14.3)
Relationship with partner	*(N =* 35)	*(N =* 6)
Spouse/legal partner	22 (62.9)	3 (50.0)
Living as married	9 (25.7)	3 (50.0)
Long‐term partner	4 (11.4)	0 (0)
Condom use with partner	*(N =* 35)	*(N =* 6)
Never/some/most of the time	28 (80.0)	6 (100)
All of the time	7 (20.0)	0 (0)
Would like to become pregnant now or as soon as possible	34 (91.9)	7 (100)

**Figure 1:**
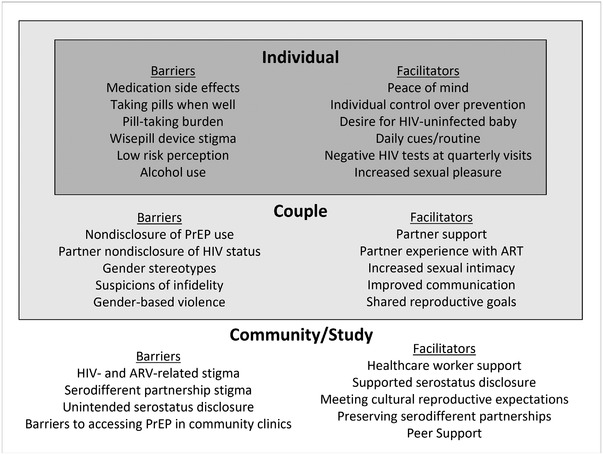
Barriers and facilitators of PrEP uptake and use among study participants.

Our qualitative data showed that PrEP use was influenced by individual, couple and community factors (Figure 1).

### 3.2 Individual level

At the individual level, side effects, taking a daily pill when well, stigma and alcohol use were described as PrEP use challenges.

*“Many people are not interested in taking these medicines every day for no good reason, especially when they don't feel sick. They say, 'If I get sick, I will swallow the drugs of HIV, but I cannot start swallowing those drugs now, yet I am not sick [HIV‐positive]. After all, I do not know if I will get infected or not.'”* – Female, age 29, High adherence (#047)

*“When I am drunk, I cannot take my medicine in the morning because I would be having a hangover and I would not feel like taking anything.”* – Female, age 28, Low adherence (#052)

However, the perceived benefits of PrEP helped some women overcome barriers.

*“… If you stop just because [PrEP's side effects have] disturbed you, then you will not achieve what you want. If you get dizzy, and vomit and say, 'It is because of this drug, I am going to stop it.' You will not achieve your desires. You have to continue taking it until the body gets used to it.”* – Female, age 19, High adherence (#065)

Motivation to meet personal goals also helped women overcome the burden of daily medication.

*“…you have to take drugs on a daily basis…Sometimes you forget, and by the time you realize you are in the market or in the bus travelling. Sometimes you have to go back home and pick your drugs or other times you miss when you are far… It is disturbing but you have to persist because you always know what you want.”* – Female, age 39, High adherence (#133)

Due to stigma of both PrEP use and HIV, some women avoided taking medication when other people were around. However, motivations to have a healthy child encouraged high adherence despite anticipated stigma associated with PrEP use.

*“Sometimes you have visitors around, and you exceed the time for swallowing [the pill] but your heart is on the drugs you know you have to swallow… I swallow to protect myself so that the child does not get a problem. I do not know if I may get infected, but I am doing this so that in future my child may not get a problem [HIV].”* – Female, age 27, High adherence (#076)

Serial HIV testing inspires PrEP confidence and motivated this woman's adherence.

*“I was scared because my husband has HIV but does not like using condoms. I decided to take PrEP to protect me and my child…whenever I go back and test and I find I am still [HIV] negative, it really motivates me. I get to know these medicines work. It is important to me so I can never forget them.” –* Female, age 25, High adherence (#075)

Women who were unsure of their partner's ART adherence behaviour and/or serostatus used PrEP to reduce their risk of HIV acquisition. PrEP restored their hope and offered a “*rare*” opportunity for them to “*get back*” individual control without depending on partners. PrEP was often initiated and used to help regain their “*peace of mind*” and offer them a desired “*double protection*.”

*“I always felt very scared because I always think that his virus is active and it may move to my blood, that is why I am taking my PrEP very well. I have no control over him, whether he takes his medicines well or not. PrEP gave me the rare opportunity to get back my power and stay away from his HIV…I know that if I continue taking my medicine well, then I don't have to worry about his virus because it will not move to my blood…It is up to me, it is in my hands.”* – Female, age 29, High adherence (#058)

“*I decided to start on my own drugs so that I do not rely on my husband's drugs because one day he may disappoint me… I have a peace of mind because even when my husband is on ARVs, I tell him we need double protection and… PrEP restored my hope of staying with him and I am very hopeful I am protected from all HIV all the time.” –* Female, age 31, High adherence (#105)

Male partners with HIV appreciated knowing their partners had added protection.

*“I am happy that you gave her PrEP because before I was worried that I may infect her with my HIV since my adherence is not very good. Now I can relax because I know that she is well protected”‐* Male, age 35, partner of #058

Women with high adherence described using cues, including television programming and phone alarms.

“*Whenever I switch on my TV, there's this program at around 9 pm that rings a bell in my ear to take my medicines*” – Female, age 27, High adherence (Participant 076)

“*I set a phone reminder. This way, I cannot forget to take my medicines”‐* Female, age 32, High adherence (#050)

### 3.3 Couple level

At the couple level, nondisclosure of PrEP use and HIV serostatus contributed to tensions, mistrust, gender stereotypes, suspicions of infidelity and gender‐based violence. This male partner articulates why women may not expect men to disclose their serostatus, even if they are planning on having a child together.

*“As you know women can never be relied on, I cannot be sure that I will stay with her… She is lucky that I agreed to test with her, if I had tested alone, I would never have told her about my HIV status.”* – Male, age 36, partner of #098

A woman whose partner has not disclosed his HIV serostatus describes why she chose to keep her PrEP use a secret.

*“I did not tell him that I am taking this medicine because he did not tell me that he has HIV, and he is on drugs. So, when I got to know I thought that I should not tell him too.”* – Female, age 34, Low adherence (#085)

The confusion between PrEP and ART could be advantageous for women whose HIV‐negative status puts their partnership at risk:

*“I could not tell him anything about PrEP because he did not know that I am HIV negative. When he saw the drugs, he knew that I had started ARVs. Even up to now he does not know that I am taking drugs which prevent me from getting infected, he knows that I am taking ARVs that treat HIV… Most times when the man finds out that the woman is sick, and he is not sick, there very high chances that he will break up with you. But when he finds out that you are not sick and he is sick and you want to leave him, he would rather kill you than letting you go. So, the good thing about PrEP is that its package, shape and size is the same as that for ARVs which is very helpful for me.”* – Female, age 40, High adherence (#125)

Some women were suspected of infidelity when their partners discovered they were using PrEP and experienced violence that drove them out of their homes, causing them to miss PrEP doses. Relationship turbulence was characterized by trust disintegration, non‐disclosure, victim blaming and emotional blackmail through attacking partner for infidelity or “*sleeping around*.”

*“After seeing him taking ARVs in hiding, I immediately went to the hospital and tested that day. I started on PrEP and kept quiet to avoid problems…he gets mad and beats me up if he gets to know I am still taking these medicines, so sometimes I hide and sometimes it was not possible because I slept out of the house after [he] beat me and locked me away so I missed taking them…he thinks I want to protect myself from getting HIV from my other numerous men, so I ran away” –* Female, age 36, Low adherence (#056)

Some women's decision not to initiate PrEP, despite eagerness for HIV prevention benefits, was driven by anticipated violence.

*“I asked my husband about his HIV‐status when we last tested together but he got very angry, shouted at me, and refused to tell me anything. So, I fear if he were to find out I came, he would say that I am swallowing HIV drugs and I have been having HIV all along. Yet he is not willing to tell me his status. It can cause chaos in my home and cause him to quarrel. I am now helpless and hopeless because I would have wanted PrEP to protect myself for sure, unlike these other methods when I am always doubtful.” –* Female, age 31, No PrEP use (#062).

For couples in mutually disclosed partnerships, PrEP was a tool in a “*shared battle*” against HIV. The safer conception program encouraged them to disclose their serostatus to each other and created a sense of restored hope to “*fight*” the virus together. Partner experiences with ART also helped women continue PrEP. This fully disclosed couple took their medications together to support daily adherence for both partners.

“*She tries to give me support in taking my drugs as she also takes hers…most times I take my drugs at 9:30 pm and we usually do that at around the same time”*. – Male, age 57, partner of #050

With less worry about HIV transmission, PrEP helped couples confidently meet their reproductive goals. The peace of mind from anticipated HIV risk reduction was reported to improve communication, intimacy, confidence and quality of relationship.

*“Ever since I started these drugs, even when am having sex, I do not have any bad thoughts, we talk all the time. I accept [sex] wholeheartedly. I do not worry about anything. I am relaxed and comfortable with him and he's very happy (Laughter). I believe the drugs will help me protect myself from HIV and have a baby free from HIV. I believe in them.” –* Female, age 25, High adherence (#075)

*“We really wanted to have a child, but she was scared about me not taking my drugs well. There was no peace at home before she started using this method [PrEP]…But now everything is good. She is relaxed, we talked about my medicines, so I am happy we are sharing this battle together. She often reminds me to take mine and at least she is also taking something now to fight the virus with me. She really looks less worried since they told her the drugs will protect her from HIV.”* – Male, age 25, partner of #075

### 3.4 Community level

Stigma related to HIV, antiretroviral drugs (ARVs) and serodifferent partnerships, and worries about unintended serostatus disclosure challenged PrEP adherence. Women worried that PrEP would mistakenly identify them as having HIV.

*“… if my friends or mother saw this medicine [PrEP] they can easily mistake it to be ARVs …that is what makes it hard for me to take them because I cannot expose myself. Most people do not know that one can take these drugs without being sick of HIV, even if I explain to them, so they begin gossiping about me and begin treating me like I am already dead.” –* Female, age 37, Low adherence (#124).

Experiences of peers seroconverting in serodifferent relationships and others using PrEP successfully inspired PrEP optimism and encouraged women to persist with PrEP use.

*“Some of my friends say that it is hard for them to take these drugs every day when they are not feeling sick, but I tell them that they would rather take it than risk their lives…As a result of that most of them now have HIV, in fact one of them even got pregnant and gave birth to a sick baby. They are now regretting and telling me that they wish they had used PrEP like other people, maybe it would have prevented them from getting HIV…so you see, I really feel lucky that I was able to get someone who has been using it [PrEP] for some time that brought me here to get this service to protect my life.” –* Female, age 29, High adherence (#047)

Women explained how PrEP use could indirectly reveal the couple's serodifferent status, and fear of being branded as “*reckless and irresponsible*” for choosing to stay with partners living with HIV led women to keep PrEP use secret, which may impact adherence.

“*I sometimes forget to carry my medicines because I did not tell anyone about my PrEP. Sometimes we have visitors so you wait until they go…The problem of sharing such things with others is that if I tell them, they will get to know that my husband has HIV…They will think I am reckless and irresponsible. They will keep telling me, 'Forget about that man, leave him, he is going to infect and kill you'. They will leave my husband no peace.” –* Female, age 26, Low adherence (#102).

PrEP efficacy perceptions and adherence were improved through ongoing support from study staff and health workers.

*“At first it [PrEP] used to make me very dizzy and I thought it was not working well on me…But I called the counsellor here and we talked a lot. He told me that I need to change the time when I take it. I was taking it in the morning, so he encouraged me to take it before going to bed, when I did that, I became very okay so now I have no problem with my medicine.”* – Female, age 31, high adherence (#109)

On study completion, women were referred to HIV care sites providing PrEP. However, women experienced or anticipated negative healthcare interactions at their community clinics and thus saw the end of the study as the end of PrEP access, suggesting a need for ongoing support for PrEP, not just at initiation.

*“I would like to ask [the doctor/counselor] questions and he gives me answers [as it was during the study] but now where I go, he does not give me the chance he just gives me the medicine and I just go.” –* Female, age 31, Low adherence (#132)

Despite fears of community stigma, using PrEP helped women meet cultural expectations to conceive while avoiding HIV infection. Women and couples face pressure from their communities to remain partnered, causing conflict when reproductive goals are not met or when couples are in an HIV‐serodifferent relationship. Women were motivated to access PrEP to avoid separation due to childlessness. PrEP offered a strategy for one couple to support their reproductive goals and maintain their partnership.

*“My husband keeps telling me that he will chase me and marry another wife that can give him a child… I always felt very scared thinking that his virus is active and can move to my blood anytime. That is why I chose PrEP because I know that if I continue taking it well, I may not get his HIV and will get a healthy baby…” –* Female, age 29, High adherence (#058)

Her partner (age 35) said,

*“For me to marry this woman, all I wanted was a child. … but things have failed to work out … my wife wants the child even more than I do because my mother keeps abusing her that she has refused to give me a child intentionally and that makes her feel very bad.”*

One couple stayed together in a serodifferent partnership to avoid negative appraisal of divorce (mainly experienced by women).

*“My husband pleaded with me to stay together and start a family despite him testing positive so I started PrEP…We just wedded last year, and I have to stay married to him, so our friends and relatives don't judge me or ask ugly questions.” –* Female, age 28, Low adherence (#098)

## 4 DISCUSSION

In this study, we aimed to understand how women in Uganda with plans for pregnancy and concerns for HIV exposure overcame barriers to achieve high uptake and adherence to daily oral PrEP. Women participating in this qualitative sub‐study articulated previously described barriers to PrEP use, such as HIV, ART and PrEP stigma, side effects, pill burden, alcohol use and couple‐level challenges around HIV serostatus and PrEP‐use disclosure, suspicions of infidelity and gender‐based violence. Factors that helped women overcome these barriers included: confidence in PrEP; peer, spousal or healthcare provider support; meeting socio‐cultural expectations to conceive and preserve serodifferent partnerships; and female‐control over HIV prevention. PrEP use improved communication, sexual intimacy and emotional support within mutually disclosed partnerships and among those unsure of partner's viral status by offering women a self‐controlled method to minimize risk. Our results, therefore, illustrate the importance of individual, couple and community factors that support women to persist with PrEP and highlight opportunities for offering PrEP to women outside of mutually disclosed serodifferent partnerships.

Our analysis emphasizes the complexity of HIV‐ and disclosure‐related stigma and the role of healthcare workers in supporting PrEP use for the duration of a woman's PrEP journey. Our data document intersections of HIV‐ and disclosure‐related stigma, risk perception, as well as gender disparities and stereotypes as factors informing consequences of male infidelity, PrEP access, uptake and continuation in a setting with differing socio‐contextual views on divorce, HIV acquisition and prevention. PrEP users face intersecting forms of stigma and stereotypes from family and community, hindering PrEP use [16, 38]. Women accessing PrEP were labelled promiscuous, sexually irresponsible or immoral [38]. Women in our study explained how disclosure of PrEP use could indirectly reveal their participation in behaviours associated with HIV (such as taking ARVs or attending an HIV care clinic), or partner's HIV serostatus, and therefore, the couple's serodifferent serostatus, igniting suspicions of infidelity, reckless sexual behaviour, gender‐based violence and other harmful community assumptions. Gender‐based violence was catalyzed by the discovery of clandestine PrEP use, especially in relationships already strained due to mistrust. In line with Calabrese, these gendered stereotypes, frustrations and stigmas manifest at multiple levels, and threatened PrEP use [38]. Future work should explore opportunities to promote PrEP use as responsible behaviour to reduce HIV prevalence instead of stigmatizing it. Although such efforts would increase the potential burden of the intervention on the healthcare system, such directed efforts could improve effectiveness in adherence support [39].

Given challenges with oral PrEP adherence, researchers are exploring opportunities to promote PrEP use at the individual level. In Kenya, an SMS intervention did not improve low adherence (27%) among young women [40]. In South Africa, adolescent girls and young women were offered cash incentives to take PrEP [41] or given standard adherence support plus drug‐level feedback [42] with little effect due to HIV‐related stigma, individual perceived benefits and difficulties accessing PrEP. Among women using PrEP in pregnancy, studies in Kenya [43] and South Africa [44] observed high PrEP uptake but low persistence (e.g. 38% returned for 1‐month refill in a study in Kenya; fewer than 50% returned over 6 months in Cape Town). In contrast, high PrEP persistence over 9 months in our study was observed and facilitated by women's perception of PrEP as a self‐controlled, effective and safe strategy for meeting cultural expectations and pressures to conceive, to have healthy HIV‐free children and minimize risk when unsure of partner's HIV serostatus or ART adherence, while improving communication and sexual intimacy among users [20]. Social support, reminders, fully disclosed partnerships and individual belief in PrEP effectiveness supported ongoing use. Our data are largely consistent with prior studies indicating that community understanding of risk, living with an HIV‐positive or undisclosed main partner [45, 46], easy access and adherence support and counselling [47] play a key role in PrEP use. The improved PrEP uptake and adherence observed in our study may have been due to provision of routine, targeted PrEP adherence support and counselling provided by the study at the clinic (an environment that could have reduced PrEP stigma or the experience thereof). In addition, our population (median age 33 years, IQR 28.1, 35.4) was older than women in studies recruiting young and adolescent women, which may have contributed to PrEP use success. Our study also documented side effects, pill burden and schedules discourage some from taking PrEP for a prolonged period, especially if they considered themselves healthy.

Although each of these 37 women were encouraged to invite partners for a separate in‐depth interview, our findings suggest that male partners who participated were those who had fully disclosed their HIV serostatus to their spouses or had discussed their concerns for HIV risk and transmission. Efforts to screen for potential relationship challenges and encourage couple disclosure through positive healthcare engagement could be important for future interventions that aim at boosting PrEP use.

Our study had a number of strengths. The analysis explored factors affecting periconception PrEP use in a cohort of women with high PrEP uptake over 9 months in rural, Southwestern Uganda, derived using inductive content analytic approach. We also sought to explore factors affecting periconception PrEP uptake and adherence among women who do not know partner status or are not necessarily in mutually disclosed partnerships. This analysis can, therefore, inform PrEP care in the context of reproductive goals. This qualitative study was part of a large mixed methods study conducted in a publicly funded and operated hospital in a rural low‐resource setting that documented high daily oral PrEP uptake prescribed over 9 months (median = 89%, IQR: 85%, 92%) [20]. Results, therefore, may have applications for similar settings especially where individuals are highly motivated to keep babies and themselves safe. Our study also had limitations. We utilized a sample size of 37 women and their spouses who enrolled in a study offering safer conception care. Participants may represent those motivated to prevent HIV infection and take PrEP.

## 5 CONCLUSIONS

Our study contributes to a greater understanding of HIV‐, serodifference‐ and disclosure‐related stigma, and the role of relationship dynamics, gender stereotypes and provider support in navigating PrEP use. Confidence in PrEP, individually controlled HIV prevention and improved partnership communication and intimacy promoted PrEP adherence. PrEP use challenges, including multiple stigmas, side effects, pill burden, alcohol use, partnership mistrust and gender‐based violence, are mediated by social, professional and partnership support. Supporting individuals to overcome context‐specific barriers to PrEP use may be an important approach to improving uptake and prolonged use. Larger studies in diverse populations could fully evaluate whether a PrEP delivery approach centred on reproductive goals facilitates uptake and persistence.

## COMPETING INTERESTS

Gilead Sciences donated TDF/FTC as PrEP for this project. However, Gilead Sciences was not involved in any part of the research work for this project. All authors declare no competing interests.

## AUTHOR CONTRIBUTIONS

ECA, MO, MBB and LTM performed the research. CP, WM, JEH, DB, MBB and LTM designed the research study. ECA, MO, MCP and LTM analysed the data. ECA wrote the first draft. All authors have read and approved the final manuscript.

## FUNDING

This research was supported by Doris Duke Charitable Foundation Clinician Scientist Development Award (PI, Matthews).

## Data Availability

The authors confirm that the data supporting the findings of this study are available within the article. Raw data that support the findings of this study are available on request from the corresponding author.
